# Copper homeostasis networks in pseudomonas aeruginosa and the organocopper metabolite fluopsin C

**DOI:** 10.1007/s10123-026-00837-w

**Published:** 2026-05-07

**Authors:** Dongwon Choi, Tariqul Islam

**Affiliations:** Department of Biological and Environmental Sciences 1, East Texas A&M University, Commerce, TX USA

**Keywords:** Copper homeostasis, Copper toxicity, *Pseudomonas aeruginosa*, Fluopsin C, Antibiotics

## Abstract

Copper is an essential redox-active trace element that serves as a catalytic cofactor in many bacterial enzymes; however, excess intracellular copper is highly cytotoxic due to mismetallation of metalloproteins and copper-driven redox cycling that promotes reactive oxygen species generation through Fenton-like chemistry. To prevent these destructive effects, bacteria maintain near-zero levels of free cytosolic copper through tightly coordinated copper homeostasis networks. This review summarizes current understanding of bacterial copper stress physiology with a focus on the exceptionally copper-tolerant opportunistic pathogen *Pseudomonas aeruginosa*. This organism deploys a multilayered detoxification strategy that includes periplasmic copper surveillance and export mediated by the CopRS–PcoBA system, as well as highly sensitive cytoplasmic copper sensing that activates CueR-regulated efflux modules. A distinctive feature of the *P. aeruginosa* copper homeostasis network is the biosynthesis of the organometallic copper complex fluopsin C via a copper-responsive biosynthetic operon and its subsequent export through a dedicated efflux pump. This pathway supports a model in which fluopsin C functions as a copper-sequestering metabolite that stabilizes Cu(I), suppresses redox-mediated toxicity, and enables safe copper removal as a ligand-bound complex. Beyond its physiological role in copper detoxification, fluopsin C exhibits broad-spectrum antibacterial and antifungal activity and pronounced cytotoxicity toward mammalian tumor cell lines, consistent with a mechanism involving membrane disruption and ionophore-like copper shuttling. These properties suggest potential utility of fluopsin C as a metal-based, membrane-active antimicrobial in localized or formulation-controlled applications. Despite renewed interest in fluopsin C, optimization of its production remains limited, underscoring the need for future cultivation and bioprocess studies to improve yield and enable broader experimental and translational evaluation.

## Biological significance of copper

Copper is a redox-active trace element essential for all known forms of life (Andrei et al. 2020; Hofmann et al. 2021; Quintana et al. 2017). It serves as a catalytic cofactor in numerous redox enzymes, including cytochrome c oxidase, superoxide dismutase, ceruloplasmin, tyrosinase, and monooxygenases (Andrei et al. 2020; Hofmann et al. 2021; Palmer and Rosenzweig 2025; Quintana et al. 2017; Sayre et al. 2000). However, excess intracellular copper is highly toxic. Copper can displace other essential metal cofactors, such as Fe and Zn, from metalloproteins (Djoko et al. 2015; Hofmann et al. 2021; Johnstone and Nolan 2015; Quintana et al. 2017; Sayre et al. 2000) and catalyze deleterious redox reactions that generate reactive oxygen species (ROS).

Most bacterial copper-binding proteins interact with copper transiently and function as copper storage proteins, chaperones, or copper-responsive regulatory proteins (Hofmann et al. 2021; Palmer and Rosenzweig 2025; Sayre et al. 2000). In contrast, cuproenzymes bind copper cofactors as integral components of their catalytic centers with much higher affinity. Some cuproenzymes bind copper with affinities approaching covalent bonds (Bandow et al. 2012; Hogarth 2012; J. T. Rubino & K. J. Franz, 2012). These enzymes participate in electron transport, monooxygenation, and superoxide dismutation reactions. Bacterial cuproenzymes are typically localized to the cytoplasmic membrane, the periplasm, or the cytoplasmic membrane surface and are generally absent from the cytosol (D.-W. Choi et al. 2014; García et al. 2012; Parmar et al. 2018; J. T. Rubino & K. J. Franz, 2012). This spatial compartmentalization of cuproenzymes minimizes copper-mediated heavy metal toxicity within the cytoplasmic space (Andrei et al. 2020; Palmer and Rosenzweig 2025; Quintana et al. 2017; Sayre et al. 2000). In addition, intracellular copper is preferentially trafficked as Cu(I)–chaperone complexes to prevent the accidental release of free copper (Hofmann et al. 2021; Palmer and Rosenzweig 2025; Jeffrey T. Rubino & Katherine J. Franz, 2012; Sayre et al. 2000). Besides, Cu(I) is poorly soluble in pH neutral aqueous environments; for example, the solubility of CuCl is approximately 47 mg/L at ambient temperature.

The near-complete absence of free copper in the bacterial cytoplasm is maintained through the coordinated action of copper chaperones, copper storage proteins, and low-molecular-weight thiol-containing compounds (Hofmann et al. 2021; Parmar et al. 2018; Scheiber et al. 2014). Given the predominance of Cu(I) and the involvement of multiple copper-trafficking molecules, intracellular copper distribution is governed by affinity gradients among cuproproteins and copper-binding compounds. These affinity gradients serve as major determinants of directional copper transfer within the cytosolic copper pool (Andrei et al. 2020; Hofmann et al. 2021; Palmer and Rosenzweig 2025; Quintana et al. 2017; Jeffrey T. Rubino & Katherine J. Franz, 2012; Sayre et al. 2000; Sesham et al. 2013).

### Fenton-like reactions

The Fenton reaction is a metal-catalyzed chemical process mediated primarily by transition metals, especially by iron(Koppenol 1993). Iron exists in two interconvertible oxidation states, ferrous ion (Fe(II) and ferric ion (Fe(III), which readily undergo redox cycling in the presence of oxidants such as hydrogen peroxide (H₂O₂) (Cai et al. 2021; Cui et al. 2014; Koppenol 1993; Wang et al. 2014). In the Fenton reaction, Fe(II) reacts with H₂O₂ to generate highly reactive hydroxyl radicals (•OH) and Fe(III) (Cai et al. 2021; Cui et al. 2014; Koppenol 1993; Wang et al. 2014). This chemical reaction has been widely utilized in industrial applications, particularly for the degradation of organic pollutants in wastewater treatment. Fenton-based processes generate potent and economically viable oxidants capable of decomposing diverse toxic compounds, including dyes, pesticides, petroleum byproducts, and pharmaceuticals (Cai et al. 2021; Koppenol 1993; Toda et al. 2014; Wang et al. 2014). In biological systems, however, the same chemistry is recognized as a major source of oxidative stress. Hydroxyl radicals are highly damaging to DNA, lipids, and proteins and have been implicated in aging and the pathogenesis of diseases such as cancer and neurodegenerative disorders (Jomova et al. 2010; Koppenol 1993; Liu et al. 2011; Wang et al. 2014).

**Fig. 1:**
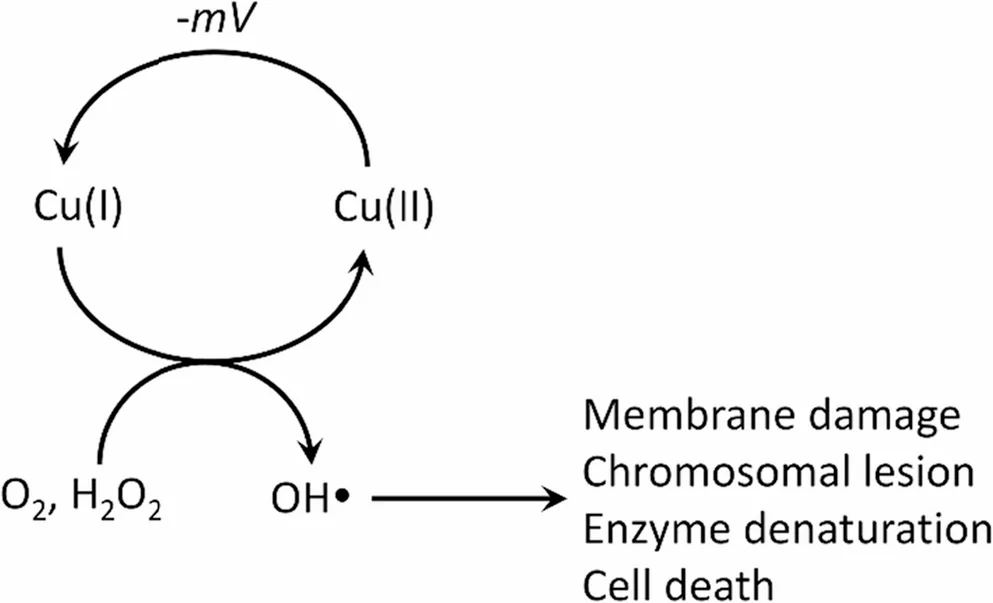
Copper-mediated Fenton-like redox cycling and reactive oxygen species generation. In the highly reducing bacterial cytoplasm, free copper undergoes continuous redox cycling between Cu(I) and Cu(II) in the presence of molecular oxygen and oxygen-derived oxidants. This process drives Fenton-like reactions that generate reactive oxygen species (ROS), including hydroxyl radicals. Elevated cytoplasmic ROS levels cause extensive damage to DNA, proteins, and lipids, ultimately leading to loss of cellular integrity and cell death.

Similar to iron, copper exists primarily in two redox states: cuprous (Cu(I) and cupric (Cu(II) (Fig. 1). The relatively high redox potential of the Cu(I)/Cu(II) couple (approximately + 160 mV) favors reactions with molecular oxygen and oxygen-derived oxidants (Fu et al. 2014; García et al. 2012; Sochocka et al. 2013). The bacterial cytoplasm is a highly reducing environment enriched in low-molecular-weight reductants such as glutathione, quinols, cysteine, and ascorbate. Under these conditions, Cu(I) predominates and readily reacts with molecular oxygen and hydrogen peroxide (Gaggelli et al. 2006; X. X. Zhang and Rainey 2008).

These reactions oxidize Cu(I) to Cu(II) while concomitantly generating hydroxyl radicals (•OH). The resulting Cu(II) is rapidly reduced back to Cu(I) by intracellular reductants, enabling repeated cycles of radical formation (Adlard and Bush 2006; Nadal et al. 2007; X. X. Zhang and Rainey 2008). This copper-mediated Fenton-like chemistry can inflict extensive cellular damage, including DNA strand breaks, protein oxidation and denaturation, lipid peroxidation, and ultimately cell death (Adlard and Bush 2006; Fu et al. 2014; Gaggelli et al. 2006) (Fig. 1).

### Copper homeostasis networks

Copper is a scarce trace element in most aquatic environments. Natural copper concentrations vary widely but typically range from 0.2 to 30 ppb in the form of cupric or cuprous ions (USEPA, 2007). Consequently, under ordinary environmental conditions, bacterial copper homeostasis networks are primarily geared toward copper acquisition due to limited copper availability in natural habitats. As a result, many bacteria lack dedicated copper tolerance mechanisms within their copper homeostasis systems (Fu et al. 2014).

Transient exposure to unusually high copper concentrations can often be tolerated through nonspecific organometallic complexation with negatively charged biomolecules located on the cell surface and within the cytoplasm. In contrast, bacteria chronically exposed to elevated copper levels have evolved specialized copper tolerance mechanisms as part of their copper homeostasis networks. Such high-copper environments include mining sites, industrial wastewater discharge areas, and agricultural soils subjected to frequent and repeated applications of copper-based fertilizers and fungicides (Cervantes and Gutierrez-Corona 1994; Cooksey 1993; Cánovas et al. 2003; Elad et al. 2010).

Host-associated environments also represent copper-rich niches. During infection, macrophages actively pump copper into phagolysosomes as an antimicrobial strategy to kill engulfed bacteria. Bacterial species that persist in these environments over extended evolutionary timescales often exhibit unusually high copper tolerance, including many plant and animal pathogens (Cha and Cooksey 1993; Cooksey 1993; Elad et al. 2010). Given the inherent toxicity of redox-active copper ions, bacterial cells must tightly regulate intracellular copper levels through coordinated copper homeostasis networks (Ghnamah et al. 2025; Hofmann et al. 2021; Palmer and Rosenzweig 2025; Sayre et al. 2000).

## Copper homeostasis network in *Pseudomonas aeruginosa*

### *Pseudomonas aeruginosa*

*Pseudomonas aeruginosa* is a Gram-negative gammaproteobacterium known for its exceptionally high copper tolerance; wild-type strains can tolerate approximately 2–4 mM Cu(II) (Abboud, Saeed, Tarawneh, Khleifat, & Al Tarawneh, 2009; Cervantes and Gutierrez-Corona 1994; Cox and Adams 1985; Egawa et al. 1970; Quintana et al. 2017; Zhang et al. 2024). It is a common member of the normal microbiota of healthy individuals, colonizing the skin, oropharynx, and gastrointestinal tract (Crone et al. 2020; Egawa et al. 1970; X. X. Zhang and Rainey 2008). However, *P. aeruginosa* is also a major opportunistic pathogen responsible for severe infections in immunocompromised hosts (Crone et al. 2020; Egawa et al. 1970; Patteson et al. 2021).

Given its long co-evolutionary history with animal hosts, *P. aeruginosa* may have developed elevated copper tolerance as an adaptation to copper-based antimicrobial defenses (Cervantes and Gutierrez-Corona 1994; Cooksey 1993; Crone et al. 2020; Hofmann et al. 2021; Quintana et al. 2017). Macrophages actively increase copper concentrations within phagosomes as part of the innate immune response, where copper functions as a potent antimicrobial agent (Cervantes and Gutierrez-Corona 1994; Cooksey 1993; Fu et al. 2014). Copper concentrations in mature phagolysosomes can reach several hundred micromolar (Andrei et al. 2020; Fu et al. 2014; Hofmann et al. 2021; Quintana et al. 2017). Consequently, the high copper tolerance of *P. aeruginosa* may confer a selective advantage by enabling partial evasion of macrophage-mediated antimicrobial activity.

### High copper tolerance of *P. aeruginosa*

The high copper tolerance of *P. aeruginosa* is mediated by a coordinated, multi-component copper detoxification strategy comprising: (i) a periplasmic copper efflux system, (ii) a cytoplasmic copper efflux system, and (iii) synthesis of copper-binding compounds that neutralize Cu(I) toxicity (Andrei et al. 2020; Ghnamah et al. 2025; Hofmann et al. 2021; Novoa-Aponte et al. 2020; Palmer and Rosenzweig 2025; Quintana et al. 2017; Sayre et al. 2000) and (iv) copper dependent transcription factors CueR and CopRS.

Under most environmental conditions, copper availability is limited, and bacteria rely on copper acquisition systems, including putative passive facilitated copper diffuser across the cytoplasmic membrane (Ghnamah et al. 2025; Hofmann et al. 2021; Sayre et al. 2000). When environmental copper concentrations increase, these uptake pathways are optimized for acquisition rather than regulation and can lead to rapid accumulation of cytoplasmic copper to toxic levels. This accumulation triggers activation of copper exportation mechanisms, collectively referred to as copper efflux systems (Cánovas et al. 2003; Hofmann et al. 2021; Novoa-Aponte et al. 2020; Palmer and Rosenzweig 2025; Parmar et al. 2018; Quintana et al. 2017; Scheiber et al. 2014).

Copper binding compounds (CBCs) are small, non-proteinaceous molecule capable of binding copper ions. CBCs involved in copper acquisition, such as methanobactin, are typically secreted in copper-free form, bind copper in the extracellular environment, and are subsequently imported through dedicated uptake pathways (D. W. Choi et al. 2010; El Ghazouani et al. 2012; Graham & Kim 2011). In contrast, CBCs involved in copper detoxification form copper complexes within the cytoplasm, and the resulting copper–ligand complexes are actively exported (Del Rio et al. 1972; Patteson et al. 2021). In *P. aeruginosa*, fluopsin C represents such a detoxification-associated copper-binding compound (Bedoya et al. 2019; Del Rio et al. 1972; Patteson et al. 2021).

Transcriptomic analyses have identified several copper-responsive genes—possibly constituting a core adaptive copper regulon (Sayre et al. 2000)—including pcoB (outer membrane porin), copA1 (Cu(I)-exporting P-type ATPase), and czcCBA (putative Cu(II) efflux system) (Andrei et al. 2020; Caille et al. 2007; Ghnamah et al. 2025; Hofmann et al. 2021; Novoa-Aponte et al. 2020; Palmer and Rosenzweig 2025; Perron et al. 2004; Rensing et al. 1997; Sayre et al. 2000). These systems are upregulated simultaneously under copper stress, suggesting that copper uptake and efflux pathways may be co-activated. Although this regulation appears counterintuitive, it likely reflects either (i) a lack of negative feedback regulation in copper uptake systems or (ii) the presence of non-selective copper entry pathways (Ghnamah et al. 2025; Palmer and Rosenzweig 2025; Quintana et al. 2017). In this model, cytoplasmic copper homeostasis is achieved primarily through tightly regulated efflux rather than controlled uptake.

#### Periplasmic copper sensing; the CopRS system

**Fig. 2:**
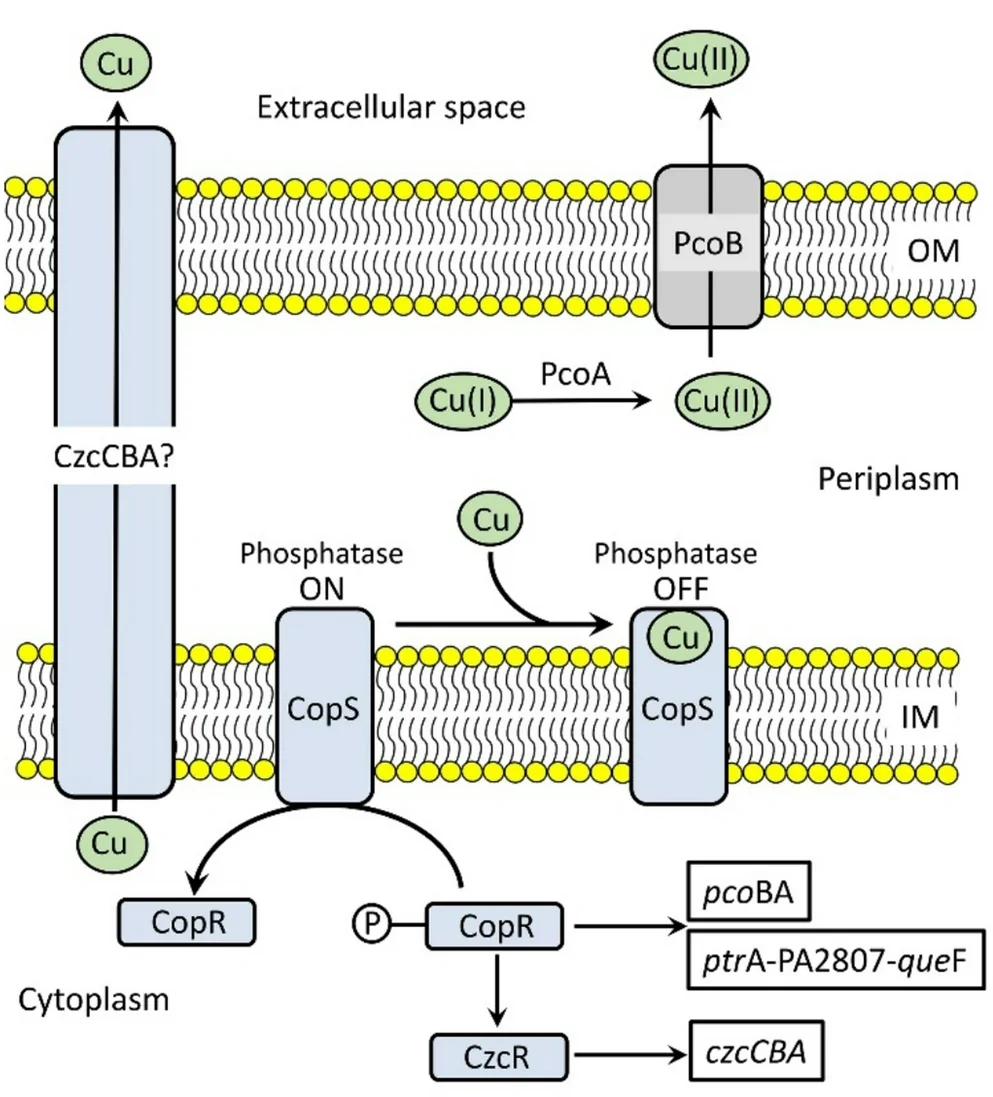
Copper efflux system mediated by the CopRS two-component regulatory pathway. The CopRS system monitors periplasmic copper levels via the periplasmic copper-sensing domain of the sensor kinase CopS. Copper binding to this domain modulates the activity of the cytoplasmic phosphatase domain of CopS, which controls the phosphorylation state of the response regulator CopR. In the absence of free periplasmic copper, CopS exhibits phosphatase activity and maintains CopR in a dephosphorylated, transcriptionally inactive state. Upon detection of free copper in the periplasm, CopS phosphatase activity is inhibited, allowing CopR to accumulate in its phosphorylated form. Phosphorylated CopR functions as an active transcriptional regulator that induces expression of the pcoBA operon, encoding the periplasmic copper efflux machinery. CopR also induces czcCBA operon. The czcCAB operon (PA2520-PA2522) encodes a transperiplasmic resistance–nodulation–division (RND)–type efflux system that spans the cytoplasmic membrane, periplasm, and outer membrane. Evidences indicate that the CzcCBA system may mediate copper ion export

The CopRS two-component system monitors periplasmic copper levels (Novoa-Aponte et al. 2020; Parmar et al. 2018; Premsuriya et al. 2023; Quintana et al. 2017; Thaden et al. 2010) (Fig. 2). It comprises the periplasmic copper sensor CopS and the cognate transcriptional regulator CopR (Novoa-Aponte et al. 2020; Thaden et al. 2010). CopS is a cytoplasmic membrane protein whose phosphatase domain faces the cytoplasm (Novoa-Aponte et al. 2020; Patteson et al. 2021; Thaden et al. 2010). Its phosphatase activity is regulated by the availability of free, uncomplexed copper ions in the periplasm (Fig. 2). When periplasmic free copper is absent, the copper-sensing domain of CopS remains unoccupied, and its phosphatase activity is active (Hofmann et al. 2021; Novoa-Aponte et al. 2020). Under these conditions, CopS maintains CopR in a dephosphorylated state. Dephosphorylated CopR is transcriptionally inactive and does not induce expression of the pcoBA operon (Hofmann et al. 2021; Novoa-Aponte et al. 2020) (Fig. 2).

When the concentration of free copper in the periplasm increases, copper binds to the sensing domain of CopS, inhibiting its cytoplasmic phosphatase activity (Hofmann et al. 2021; Novoa-Aponte et al. 2020; Sayre et al. 2000). As a result, the intracellular level of phosphorylated CopR increases. Phosphorylated CopR functions as an active transcriptional regulator and induces expression of multiple genes including the pcoBA operon within the intracellular copper homeostasis network (Hofmann et al. 2021; Novoa-Aponte et al. 2020; Quintana et al. 2017) (Fig. 2).

The pcoBA operon encodes a periplasmic copper efflux system comprising the outer membrane copper-exporting porin PcoB and the multicopper oxidase PcoA. Owing to the reducing environment at the cytoplasmic membrane surface and within the periplasm, most periplasmic copper exists in the cuprous (Cu(I) state. Because Cu(I) is poorly soluble in near neutral aqueous environments, its oxidation to the more soluble cupric ion (Cu(II) oxidized by PcoA facilitates efficient copper export to the extracellular space (Andrei et al. 2020; Ghnamah et al. 2025; Hofmann et al. 2021; Palmer and Rosenzweig 2025; Quintana et al. 2017; Jeffrey T. Rubino & Katherine J. Franz, 2012) (Fig. 2).

CzcRS system is a two-component regulatory system in *P. aeruginosa* controlling cadmium, zinc, and cobalt (hence czc) resistance by inducing the CzcCBA proton dependent cation antiport (Caille et al. 2007; Rensing et al. 1997). The system is cross-regulated by copper stress through the CopRS regulatory system (Caille et al. 2007). The czcCBA gene expression is strongly upregulated by high copper conditions (10mM), with reported induction level ranging from 62 to 390fold, yet only marginal effects on copper resistance have been observed (Teitzel et al. 2006; Thaden et al. 2010).

Comparative analyses further highlight the differential distribution of copper efflux systems. Disruption of copA (PA3920), encoding a Cu(I)-specific P-type ATPase, results in substantially increased copper sensitivity at relatively low concentrations (1–2 mM), whereas the czcC mutant exhibits no appreciable sensitivity until exposure to ~ 8 mM copper (Teitzel et al. 2006). These observations suggest that (i) CopA functions as the primary copper efflux system, (ii) CopA alone may be insufficient under high copper stress conditions (≥ 8 mM), and (iii) CzcCBA may function as a secondary or auxiliary efflux system under extreme copper loads.

However, this interpretation is complicated by studies reporting that czcA mutants do not exhibit reduced copper tolerance at 6–8 mM copper (Caille et al. 2007; Perron et al. 2004), arguing against a direct role of this system in copper detoxification. Additionally, reported copper tolerance levels for wild-type *P. aeruginosa* PAO1 vary considerably across studies. Teitzel et al. (2006) observed near-normal growth up to ~ 10 mM copper, with complete inhibition at ~ 16 mM, whereas other studies reported complete growth inhibition at ~ 6 mM (Caille et al. 2007; Perron et al. 2004).

To address this discrepancy, we conducted cultivation assays across a range of copper concentrations using wild-type *P. aeruginosa* PAO1. Our results are consistent with those of Teitzel et al., showing minimal inhibition at ~ 10 mM and complete growth inhibition at ~ 15 mM copper. Increased sensitivity became noticeable only at ≥ 8 mM copper (data not shown). These findings support a model in which additional efflux systems, such as CzcCBA and possibly MexPQ–OpmE, contribute to copper tolerance only when intracellular copper levels exceed the capacity of the primary CopA-mediated efflux system.

CzcCBA is a well-characterized resistance–nodulation–division (RND) type heavy metal efflux system known to export Co(II), Zn(II), and Cd(II). Functional studies have demonstrated a stepwise increase in resistance depending on complex assembly: basal resistance with CzcA (cytoplasmic membrane transporter) alone, increased resistance with CzcA plus the C-terminal domain of CzcB (membrane fusion protein), further enhancement with inclusion of CzcC, and maximal resistance with the fully assembled CzcCBA complex (Rensing et al. 1997). Given that czcA deletion does not significantly affect copper tolerance up to ~ 8 mM, it is possible that partial assemblies such as the CzcCB subcomplex retain residual efflux activity in periplasmic space, potentially contributing to metal homeostasis under specific conditions.

#### Cytoplasmic copper sensing: the CueR system

**Fig. 3:**
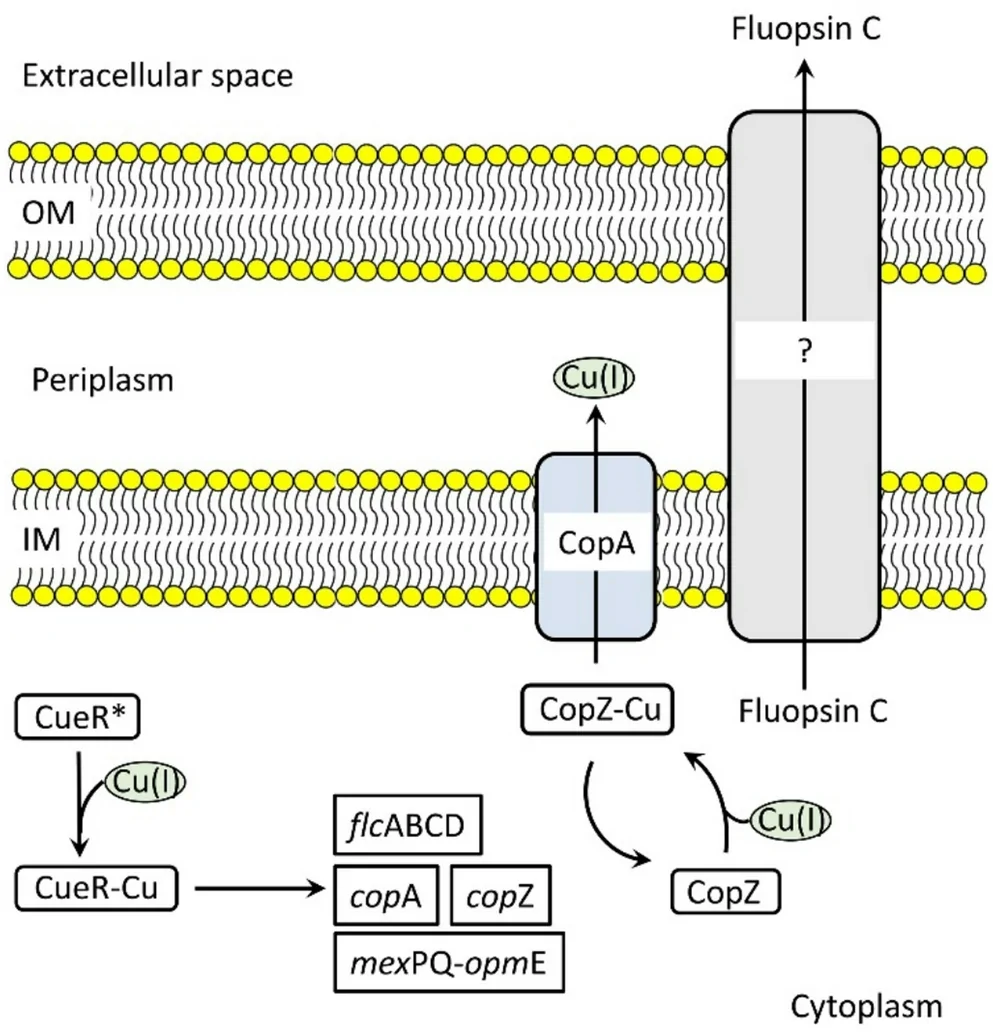
**C**opper efflux system regulated by CueR. Cytoplasmic copper stress is sensed by the Cu(I)-responsive transcriptional regulator CueR. Upon Cu(I) binding, CueR activates expression of multiple genes involved in copper detoxification, including *flc*ABCDE (PA3515-PA3519), *cop*A, *cop*Z, and *mex*PQ–*opm*E (PA3521-PA3523). The *flc*ABCDE operon encodes enzymes required for the biosynthesis of the copper-binding metabolite fluopsin C, whereas *mex*PQ–*opm*E encodes the efflux system potentially responsible for fluopsin C export. However, this possibility has to be experimentally validated. CopA functions primarily in cytoplasmic copper detoxification and receives Cu(I) from the high-affinity cytoplasmic chaperone CopZ. CopZ serves a dual role by transiently buffering Cu(I) to mitigate acute copper stress while delivering excess copper to CopA for ATP-dependent export across the cytoplasmic membrane

The cytoplasmic copper-sensing system operates independently of the periplasmic sensing system, allowing *P. aeruginosa* to prevent copper toxicity within these two distinct cellular compartments (Andrei et al. 2020; Ghnamah et al. 2025; Novoa-Aponte et al. 2020; Palmer and Rosenzweig 2025; Sayre et al. 2000) (Fig. 3). The cytoplasmic copper-sensing system is substantially more sensitive than the periplasmic system: whereas periplasmic copper sensors respond to femtomolar copper concentrations, cytoplasmic copper sensors respond to sub-attomolar levels of free copper (Hofmann et al. 2021; Novoa-Aponte et al. 2020; Parmar et al. 2018; Sayre et al. 2000). Cytoplasmic copper-responsive transcription factor binds Cu(I) with dissociation constants in the sub-attomolar range, ensuring that free cytosolic copper remains virtually undetectable (Ghnamah et al. 2025; Hofmann et al. 2021; Palmer and Rosenzweig 2025; Sayre et al. 2000) (Fig. 3).

In *P. aeruginosa*, the quorum-sensing regulator LasR directly activates a regulatory pathway involved in resistance to copper toxicity and elevated intracellular copper levels (Andrei et al. 2020; Ghnamah et al. 2025; Palmer and Rosenzweig 2025; Quintana et al. 2017; Sayre et al. 2000). Copper activates LasR, which in turn induces expression of CueR. CueR is a Cu(I)-responsive transcriptional regulator that senses cytoplasmic copper stress. Upon activation, CueR induces transcription of at least 11 genes, including flcABCDE (PA3515-PA3519), copA, copZ, and mexPQ-opmE (PA3521-PA3523) (Teitzel et al. 2006; Thaden et al. 2010)(Fig. 3). The flcABCDE operon encodes enzymes required for the biosynthesis of fluopsin C, while mexPQ-opmE may encodes a putative efflux system potentially responsible for fluopsin C export.

Resistance–nodulation–division (RND) family transporters are widespread in Gram-negative bacteria and export a broad range of cytotoxic substrates, including antibiotics and heavy metal ions. These RND efflux transporter systems are driven by the proton motive force (Andrei et al. 2020; Hofmann et al. 2021; Palmer and Rosenzweig 2025; Quintana et al. 2017; Sayre et al. 2000). *Pseudomonas aeruginosa* PAO1 encodes 11 RND genes including mexAB-oprM (PA425-427) (X. Z. Li et al. 1995), mexCD-oprJ (PA4597-PA4599) (Poole et al. 1996), mexEF-oprN (PA2493-PA2495) (Köhler et al. 1997), mexXY (PA2018-PA2019)(Masuda et al. 2000; Morita et al. 2012), mexJK (PA3676-PA3677)(Amieva et al. 2022; Chuanchuen et al. 2002), mexHI-opmD (PA4206-PA4207) (Sekiya et al. 2003), mexPQ-opmE (PA3521-PA3523) (Mima et al. 2005; Thaden et al. 2010), mexVW (PA4374-4375) (Li et al. 2003), muxABC-opmB (PA2525-2528) (Mima et al. 2009), czcCBA (PA2520-PA2522)(Caille et al. 2007; Ducret et al. 2020; Rensing et al. 1997), and a putative RND efflux transporter (PA1435-PA1436) (Lewenza et al. 2005). In a previous study (Teitzel et al. 2006), three RND systems were upregulated by copper stresses; either following sudden exposure to 10mM copper or during growth in 10mM copper amended media. Three RND efflux transporter genes include mexPQ-opmE (PA3523-174 fold, PA3522-66 fold, PA3521-460 fold expression), czcCBA (PA2522-62 fold, PA2521-118 fold, PA2520-390 fold expression increase), and the putative RND efflux transporter gene (PA1435-12 fold, PA1436-23 fold expression increase) were upregulate by the copper stress. These finding strongly suggested that these three RND efflux systems are actively responding to copper stresses in *P. aeruginosa* and may play a pivotal role in the copper efflux system within the copper homeostasis network. MexPQ–OpmE (PA3521–PA3523) was initially misannotated as a CusCAB-like efflux system due to sequence similarity to the CusCFBA system in *Escherichia coli* (Quintana et al. 2017). However, subsequent studies clarified that this locus encodes the MexPQ–OpmE efflux system (Hofmann et al. 2021). The mexPQ-opmE operon encodes a transperiplasmic multidrug efflux system spanning the cytoplasmic membrane, periplasm, and outer membrane (Fig. 3). MexQ is an integral cytoplasmic membrane protein that interacts with the periplasmic adaptor protein MexP, while OpmE is an outer membrane channel protein. Together, these components form a continuous efflux conduit. While MexPQ–OpmE is not established as a direct copper transporter, it may contribute to copper tolerance by exporting copper-associated compounds, such as fluopsin C, thereby facilitating indirect copper efflux (Fig. 3).

CopA is a Cu(I)-specific P-type ATPase. P-type ATPases constitute a large family of ATP-dependent transmembrane transporters that pump ions against electrochemical gradients through the formation of transient phosphorylated intermediates(Ducret et al. 2020; González-Guerrero et al. 2010; Kaplan 2002; Toyoshima and Inesi 2004). In *P. aeruginosa*, two CopA paralogs have been identified. CopA1 functions primarily in copper detoxification and receives Cu(I) from the cytoplasmic chaperone CopZ. CopZ is a universally conserved Cu(I)-binding protein with an exceptionally high affinity for copper (binding constants on the order of 10⁻²² M), accounting for the near absence of free copper in the bacterial cytosol(Wijekoon et al. 2015; X. X. Zhang and Rainey 2008). CopZ likely serves a dual role by acting as a transient Cu(I) storage pool that enables rapid buffering of copper stress while simultaneously delivering excess Cu(I) to CopA1 for export across the cytoplasmic membrane (Hofmann et al. 2021; Quintana et al. 2017). Genes encoding CopZ are frequently co-localized with copA and copper-responsive transcriptional regulators (Andrei et al. 2020; Ghnamah et al. 2025; Hofmann et al. 2021; Quintana et al. 2017) (Fig. 3).

CopA1-mediated export may be functionally coupled to the MexPQ-OpmE efflux system, facilitating complete removal of Cu(I) from the cell. In contrast, CopA2 is primarily involved in cuproprotein biogenesis under low-copper conditions. Together, these observations indicate that CopA1 is the dominant copper efflux ATPase during high copper stress (Andrei et al. 2020; Hofmann et al. 2021; Quintana et al. 2017; Sayre et al. 2000; Teitzel et al. 2006) (Fig. 3).

#### Fluopsin C biosynthesis

Copper-binding organometallic compounds are synthesized by bacteria for both metal acquisition and sequestration of toxic heavy metals (Andrei et al. 2020; Cánovas et al. 2003; Palmer and Rosenzweig 2025; Quintana et al. 2017). *P. aeruginosa* produces the copper-chelating compound fluopsin C (copper bis(N-methyl-N-oxidomethanethioamide); C₄H₈CuN₂O₂S₂; MW 243.8) in response to elevated cytoplasmic copper stress (Del Rio et al. 1972; Ma et al. 2013; Patteson et al. 2021).

The biosynthetic pathway of fluopsin C has been previously characterized (Patteson et al. 2021). A putative gene cluster comprising PA3515–PA3519, which is under the control of the CueR regulon, has been identified as a single operon, flcABCDE, encoding five enzymes required for fluopsin C biosynthesis (Andrei et al. 2020; Patteson et al. 2021; Quintana et al. 2017).

**Fig. 4:**
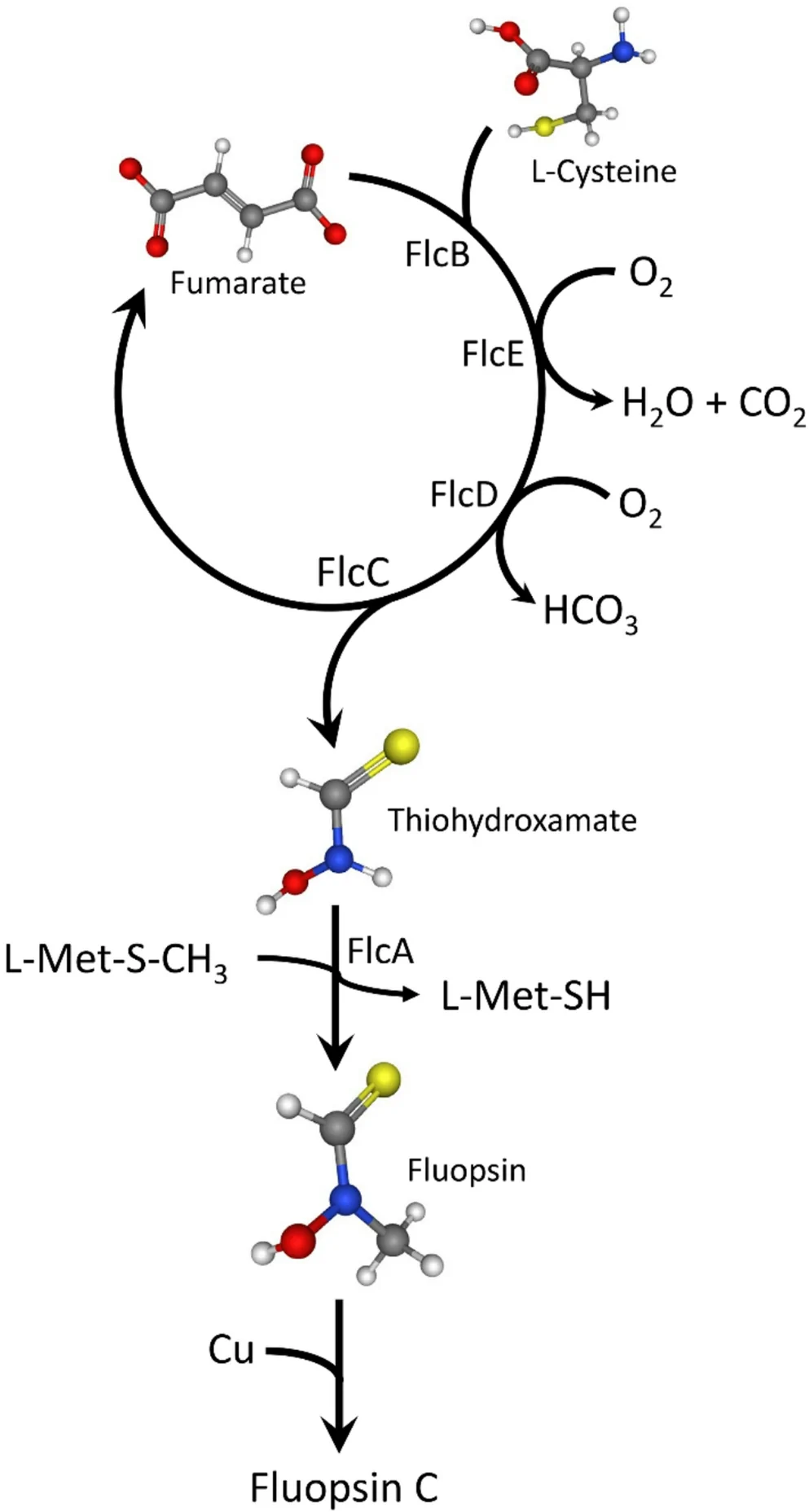
Biosynthesis pathway of Fluopsin C. The biosynthetic pathway of fluopsin C was previously characterized by Patteson et al. (2021). In this pathway, FlcB initiates fluopsin C biosynthesis by catalyzing the conjugate addition of L-cysteine to fumarate. The resulting cysteinyl adduct is subsequently processed by FlcE, an Fe(II)-dependent enzyme that mediates oxidative decarboxylation and N-hydroxylation. FlcD, another Fe(II)-dependent enzyme containing a heme oxygenase–like domain, catalyzes methyl excision as formate via an oxygen-dependent four-electron oxidation reaction. FlcC then functions as a lyase, cleaving the C–S bond to remove the S-succinyl group and generate a thiohydroxamate intermediate and fumarate. The released fumarate is recycled as a substrate for the FlcB-catalyzed conjugate addition reaction. Finally, the thiohydroxamate intermediate is methylated by FlcA, a S-adenosyl-L-methionine–dependent methyltransferase, yielding copper-free fluopsin (N-methyl-N-oxidomethanethioamide). In the terminal step, two fluopsin molecules coordinate a Cu(I) ion to form the active fluopsin C complex

FlcB is the first enzyme in the fluopsin C biosynthetic pathway and catalyzes the conjugate addition of L-cysteine to fumarate (Patteson et al. 2021) (Fig. 4). The subsequent reaction is catalyzed by FlcE, an Fe(II)-dependent enzyme that mediates oxidative decarboxylation and N-hydroxylation of the cysteinyl adduct (Patteson et al. 2021). FlcD, another Fe(II)-dependent enzyme containing a heme oxygenase–like domain, catalyzes methyl excision as formate through an oxygen-dependent four-electron oxidation reaction (Patteson et al. 2021) (Fig. 4).

FlcC functions as a lyase that catalyzes C–S bond cleavage, removing the S-succinyl group to generate thiohydroxamate and fumarate (Patteson et al. 2021). The released fumarate is recycled to serve as a substrate for the initial FlcB-catalyzed conjugate addition reaction (Patteson et al. 2021). The thiohydroxamate intermediate then undergoes methylation by FlcA, a S-adenosyl-L-methionine–dependent methyltransferase, yielding Cu-free fluopsin (N-methyl-N-oxidomethanethioamide) (Patteson et al. 2021) (Fig. 4).

In the final step of the pathway, two fluopsin molecules coordinate a Cu(I) ion to form the active fluopsin C complex(Patteson et al. 2021). Due to the highly reducing environment of the bacterial cytosol, Cu(I) is the predominant copper species available for coordination(Andrei et al. 2020; Hofmann et al. 2021; Patteson et al. 2021; Quintana et al. 2017) (Fig. 4).

#### Function of fluopsin C

The presence of free copper ions in the cytoplasmic space is highly hazardous to cellular life. Copper can displace essential metal cofactors such as Zn, Fe, and Ni from metalloproteins, as predicted by the Irving–William’s series (Beswick et al. 1976; Cervantes and Gutierrez-Corona 1994; Cooksey 1993; Ellingsen et al. 2007; Hofmann et al. 2021; Jones et al. 2007; Stadtman 1990). In addition, free copper catalyzes the formation of reactive oxygen species (ROS), which are associated with protein oxidation, lipid peroxidation, and cellular damage (Bandow et al. 2012; Cervantes and Gutierrez-Corona 1994; Cooksey 1993; Multhaup et al. 1997). To mitigate the toxic effects of cytoplasmic copper, bacterial cells employ multiple layers of protective mechanisms, including copper storage proteins, copper chaperones, and high-affinity copper-binding proteins (Andrei et al. 2020; Bandow et al. 2012; Cervantes and Gutierrez-Corona 1994; Cooksey 1993; Hofmann et al. 2021; Palmer and Rosenzweig 2025). These responses are coordinated through the copper responsive two component regulatory systems such as CueRS or CopRS, and possibly CzcRS. Some bacteria possess an additional protective strategy through the biosynthesis of small copper-binding organic compounds (Palmer and Rosenzweig 2025) called copper binding compounds (CBCs).

*P. aeruginosa* synthesizes the organocopper compound fluopsin C in response to elevated intracellular copper stress (Bandow et al.; Ghnamah et al. 2025; Hofmann et al. 2021; Palmer and Rosenzweig 2025; Patteson et al. 2021). Activation of fluopsin C biosynthesis is regulated by the cytoplasmic copper-sensing transcription factor CueR (Bandow et al. 2012; Hofmann et al. 2021; Patteson et al. 2021; Quintana et al. 2017). The cyclic biosynthetic pathway utilizes fumarate as a molecular carrier to mediate successive two-carbon removal reactions from L-cysteine, generating a thiohydroxamate intermediate that subsequently undergoes methyl transfer to form fluopsin (Palmer and Rosenzweig 2025; Patteson et al. 2021). In fluopsin C, two fluopsin molecules coordinate a single Cu(I) or Cu(II) ion through a bidentate 2O/2S ligand system (Egawa et al. 1970; Ito et al. 1971; Palmer and Rosenzweig 2025; Patteson et al. 2021).

In general, copper-binding sites in proteins are enriched in histidine, cysteine, and methionine residues, consistent with the Hard and Soft Acids and Bases principle (HSAB) (Chassaing et al. 2012; Huffman and O’Halloran 2001; Hureau 2012; Jeffrey T. Rubino & Katherine J. Franz, 2012). According to HSAB theory, Cu(I) is classified as a soft acid, whereas Cu(II) is a borderline acid. As a soft acid, Cu(I) preferentially binds ligands with large, polarizable donor atoms of low electronegativity (Huffman and O’Halloran 2001; Jeffrey T. Rubino & Katherine J. Franz, 2012). Accordingly, protein copper-binding sites frequently involve sulfur- and nitrogen-containing amino acids such as cysteine, methionine, and histidine (D.-W. Choi et al. 2014; Marino et al. 2007; Jeffrey T. Rubino & Katherine J. Franz, 2012).

Copper coordination geometry is also strongly dependent on oxidation state. Cu(I) typically adopts linear, trigonal planar, or tetrahedral coordination geometries involving two, three, or four ligands, respectively (Bandow et al. 2012; Jeffrey T. Rubino & Katherine J. Franz, 2012). In contrast, Cu(II) commonly exhibits square planar, square pyramidal, or distorted octahedral geometries with four to six coordinating ligands (Huffman and O’Halloran 2001; Jeffrey T. Rubino & Katherine J. Franz, 2012). Many redox-active copper sites that cycle between Cu(I) and Cu(II), including those found in cuproenzymes, prefer tetrahedral coordination (Huffman and O’Halloran 2001; Quintana et al. 2017; Jeffrey T. Rubino & Katherine J. Franz, 2012). This geometry imposes structural strain on the Cu(I) state, elevating its redox potential and thereby enhancing catalytic reactivity (Huffman and O’Halloran 2001; Jeffrey T. Rubino & Katherine J. Franz, 2012).

While Cu(I)/Cu(II) redox cycling is essential for the catalytic activity of cuproenzymes, copper-binding proteins and organocopper compounds involved in intracellular copper trafficking must avoid such redox cycling to prevent toxicity (Andrei et al. 2020; Hofmann et al. 2021; Quintana et al. 2017; Sayre et al. 2000). These systems require copper-binding motifs with more negative reduction potentials to stabilize the Cu(I) oxidation state. Strong electron-donating ligands, particularly those rich in sulfur and nitrogen atoms, provide such stabilization (Andrei et al. 2020; Quintana et al. 2017; Jeffrey T. Rubino & Katherine J. Franz, 2012). Coordination environments enriched in sulfur donors create a thermodynamically favorable setting that suppresses undesirable Cu(I)/Cu(II) redox cycling.

**Fig. 5:**
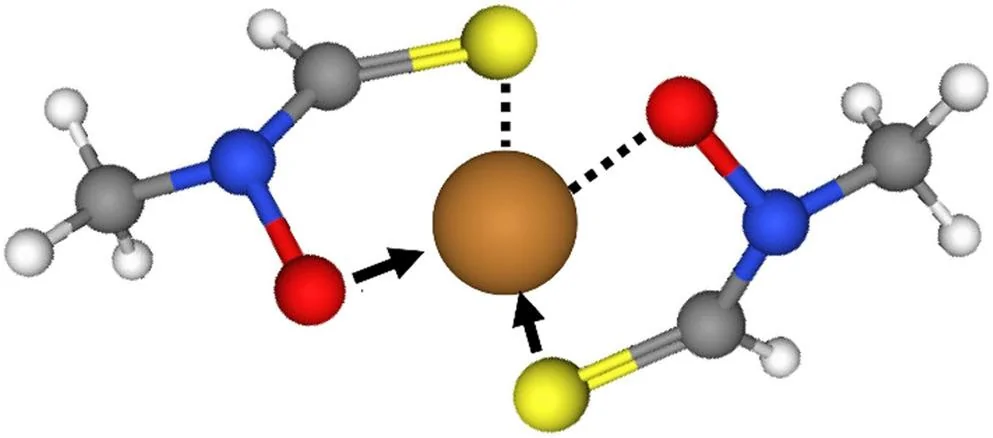
Structure of Fluopsin C. Fluopsin C is a copper-containing organometallic complex composed of two fluopsin ligands coordinated to a central copper ion (C_2_H_5_NOS-Cu(II)-C_2_H_5_NOS). Two fluopsin molecules coordinate the copper center through a mixed 2 S/2O ligand environment in a square planar geometry. This coordination stabilizes the reduced copper state and makes oxidation energetically unfavorable under the reducing conditions of the cytoplasm. The copper ion (orange) is coordinated by 2 sulfur (yellow) 2 oxygen (red) ligation. Atoms are color coded as Carbon (gray), Hydrogen(white), and Nitrogen (blue). Expected bonding distance Cu-O, 1.9–2.0.9.0Å; Cu-S, 2.2–2.3Å

Fluopsin C coordinates Cu(I) through a mixed 2 S/2O ligand environment in a square planar geometry (Fig. 5). This coordination stabilizes the reduced copper state and makes oxidation energetically unfavorable under the reducing conditions of the cytoplasm (Andrei et al. 2020; Hofmann et al. 2021; Palmer and Rosenzweig 2025; Quintana et al. 2017; Jeffrey T. Rubino & Katherine J. Franz, 2012; Sayre et al. 2000). This coordination chemistry effectively neutralizes copper toxicity by preventing redox-mediated reactivity while permitting safe intracellular copper sequestration.

Fluopsin C functions as a copper-sequestering molecule that mitigates cytoplasmic copper toxicity (Andrei et al. 2020; Patteson et al. 2021; Quintana et al. 2017; Sayre et al. 2000). Its sulfur- and oxygen-rich coordination environment stabilizes Cu(I), reducing its redox activity and minimizing ROS generation. This stabilization aligns with Hard–Soft Acid–Base principles, whereby soft sulfur donors preferentially bind Cu(I), lowering its redox potential and preventing harmful cycling between Cu(I) and Cu(II).

#### Fluopsin C transportation

For the optimization of Fluopsin C bioproduction, characterization of Fluopsin C exportation system is essential. However, the extracellular transportation system has not been experimentally validated. *P. aeruginosa* PAO1 encodes 11 RND efflux system genes as explained in Sect. 2.2.2. Among these, three RND transporter genes were upregulated by copper stresses; either following sudden exposure to 10mM copper or during growth in 10mM copper amended media. These RND efflux transporter genes include mexPQ-opmE (66-to 460-fold upregulation), czcCBA (62-to 390-fold upregulation), and a putative RND efflux transporter gene (12- to 23-fold upregulation). Since mexPQ-opmE and czcCBA showed particularly strong induction, both were considered candidates for involvement in the copper homeostasis network. However, CzcCBA system has been characterized as a cation efflux pump for heavy metal ions, CzcCBA appears unlikely to be a fluopsin C exporter. This leaves MexPQ-OpmE as a strong candidate for fluopsin C efflux system.

Nevertheless, MexPQ–OpmE efflux pump has also been predicted not to participate directly in copper homeostasis in *P. aeruginosa*. This interpretation was based primarily on the absence of methionine residues or other metal-coordinating ligands in the MexQ subunit that would typically be required for Cu(I) binding sites (Hofmann et al. 2021). MexPQ–OpmE is classified as a resistance-nodulation-division (RND)–type multidrug efflux pump, a family in which several members mediate heavy-metal export through methionine-rich metal-binding sites (Mima et al. 2005; Quintana et al. 2017; X. X. Zhang and Rainey 2008). The lack of these conserved metal-binding motifs in MexPQ–OpmE led to the suggestion that this system does not export copper ions and therefore may not contribute to copper homeostasis in *P. aeruginosa*.

However, other studies have proposed marginal contribution of MexPQ–OpmE in copper detoxification (Teitzel et al. 2006; Thaden et al. 2010). Accordingly, uncertainty remains regarding whether MexPQ–OpmE indirectly contributes to copper export. Rather than transporting free Cu(I) ions, MexPQ–OpmE may export Cu(I) complexed within fluopsin C ([Fluopsin]_2_-Cu(I). This hypothesis is consistent with the known function of MexPQ–OpmE as a multidrug efflux pump with substrate preference for hydrophobic planar organic compounds such as macrolides and fluoroquinolones (Mima et al. 2005). Fluopsin C is a planar organocopper antibiotic molecule (Del Rio et al. 1972; Novoa-Aponte et al. 2020), and its biosynthetic operon, flcABCDE, is co-regulated with mexPQ-opmE under the control of the CueR regulon (Novoa-Aponte et al. 2020; Patteson et al. 2021). Additional indirect evidence supports this model. Deletion of mexPQ results in increased copper sensitivity in *Pseudomonas aeruginosa* PAO1 (Thaden et al. 2010), suggesting a role in copper tolerance despite the absence of canonical Cu(I)-binding motifs in MexPQ. Taken together, these observations support a hypothetical model in which MexPQ–OpmE contributes to copper homeostasis by exporting [fluopsin]_2_–Cu(I) complexes rather than free copper ions. This possibility, however, requires further experimental validation, especially under high copper stress conditions (~ 10mM).

## Antibiotic properties of fluopsin C

Fluopsin C exhibits broad-spectrum antimicrobial activity. Both Gram-positive and Gram-negative bacteria are sensitive to fluopsin C, and the compound also displays toxicity toward several fungal species (Del Rio et al. 1972; Egawa et al. 1970; Ito et al. 1971; Otsuka, Niwayama, Tanaka, Take, & Uchiyama, 1972). In addition to its antimicrobial properties, fluopsin C has demonstrated pronounced cytotoxic effects against multiple cancer cell lines in vitro (Ma et al. 2013).

### Antitumor activities

A tumor is an abnormal mass of tissue resulted from uncontrolled cell proliferation. While tumors may be benign, malignant tumors acquire the ability to invade surrounding tissues and metastasize to distant organs, at which point they are classified as cancers (J. Yu et al., 2020). Cancer comprises a heterogeneous group of diseases characterized by unregulated proliferation of neoplastic cells and remains a leading cause of mortality worldwide (Ma et al. 2013; J. Yu et al., 2020).

Modern cancer treatment employs a tri-pronged strategy consisting of surgery, radiation therapy, and chemotherapy (J. Yu et al., 2020). Chemotherapy relies on a wide range of chemical agents derived from natural or synthetic sources, including alkylating agents, antimetabolites, mitotic inhibitors, topoisomerase inhibitors, and antitumor antibiotics. Antitumor antibiotics, many of which originate from soil microorganisms, represent a clinically important class of chemotherapeutic agents (J. Yu et al., 2020). These compounds exert their effects through mechanisms such as DNA damage, inhibition of protein synthesis, or disruption of cell cycle progression, ultimately leading to growth arrest or cell death of cancer cells (Ma et al. 2013; J. Yu et al., 2020).

Fluopsin C has been recognized as a potential antitumor antibiotic (Del Rio et al. 1972; Egawa et al. 1970; Egawa et al., 1971; Ito et al. 1971; Ma et al. 2013). It was originally designated YC-73 by Japanese researchers who first isolated and characterized antibiotic organocopper compounds from the culture supernatant of *Pseudomonas reptilivora* in 1970s(Del Rio et al. 1972; Egawa et al. 1970; Egawa et al., 1971; Ito et al. 1971). YC-73 was later isolated independently from *Streptomyces* spp. as grayish-green crystalline needles and identified as an antitumor compound with a minimal inhibitory concentration of 0.195 µg/mL against HeLa cells in vitro (Otsuka et al., 1972). Despite its potent biological activity, fluopsin C received limited attention for several decades due to its characteristic nonspecific cytotoxicity, as it exhibits toxicity toward cells from both the domains Bacteria and Eukarya, limiting its therapeutic applicability.

As part of natural evolutionary processes, cancer cells frequently develop resistance to antitumor antibiotics through mechanisms such as increased efflux, enhanced DNA repair, structural modification of molecular targets, and suppression of apoptosis (Alves de Lima et al., 2022; Cao et al., 2024; K. L. Yu & Shen, 2025). The emergence of drug-resistant cancers has renewed interest in alternative antitumor agents, leading to the recent re-evaluation of fluopsin C (Alves de Lima et al., 2022; Ma et al. 2013; Navarro et al., 2019; Patteson et al. 2021).

Recent studies demonstrated strong antitumor activity of fluopsin C against human breast adenocarcinoma cell lines MCF-7 and MDA-MB-231(Alves de Lima et al., 2022; Ma et al. 2013). Mechanistic analysis revealed that fluopsin C induces cytotoxicity primarily through oncosis (Ma et al. 2013). Oncosis is a form of non-programmed cell death characterized by cellular swelling, increased membrane permeability, and eventual membrane rupture (Ma et al. 2013; J. Yu et al., 2020). Another study reported concentration-dependent cytotoxicity of fluopsin C against human mammary tumor cell lines and mammary epithelial cells, with IC₅₀ values of approximately 1 µM and 2.4 µM, respectively (Alves de Lima et al., 2022). Unlike apoptosis, which is often mediated by mitochondrial ROS signaling, oncosis is typically associated with disruption of plasma membrane integrity. Together, these findings suggest that fluopsin C may function as an ionophore targeting the cytoplasmic and possibly mitochondrial membranes. The potential mechanisms include: (i) partitioning of hydrophobic ionophores into the lipid bilayer, which alters lipid packing and membrane fluidity and thereby compromises barrier function; (ii) carrier-mediated ion transport by fluopsin C, resulting in dissipation of electrochemical gradients and subsequent energetic failure; and (iii) promoting intracellular copper ion accumulation and subsequent redox cycling and ROS production.

### Antifungal activities

*Candidozyma auris* (formerly called *Candida auris*) is an emerging fungal pathogen responsible for candidiasis and invasive systemic infections, particularly in immunocompromised individuals (Navarro et al., 2019). It is a persistent skin colonizer in long-term care facilities and is classified as a priority pathogen due to its multidrug resistance (Navarro et al., 2019; Spoladori et al., 2023).

Fluopsin C, together with 3-indolinone (C₈H₇NO), was identified in a semi-purified fraction (F4a) derived from a low-virulence *P. aeruginosa* LV strain isolated from citrus canker lesions on Citrus sinensis (Bedoya et al. 2019). The bacterium was cultured in nutrient broth supplemented with 0.01% CuCl₂·2 H₂O to induce fluopsin C production under copper stress. The F4a fraction was obtained from culture supernatants using dichloromethane extraction followed by silica gel flash chromatography (Bedoya et al. 2019; Navarro et al., 2019). The fluopsin C–containing F4a fraction exhibited fungicidal activity against planktonic *C. auris* cells. When combined with silver nanoparticles, F4a displayed enhanced fungicidal effects, causing extensive cellular damage at concentrations that were non-toxic to mammalian cells (Bedoya et al. 2019; Navarro et al., 2019; Spoladori et al., 2023).

### Antibacterial activities

Biological membranes function as selective permeability barriers that regulate the exchange of ions and molecules between the cytoplasm and the external environment. Maintenance of membrane integrity is essential for cellular homeostasis. Ionophores are a class of antimicrobial agents that disrupt membrane function by facilitating uncontrolled ion transport across lipid bilayers, leading to membrane depolarization and ionic imbalance (Ding and Lind 2009; Kevin Ii, Meujo, & Hamann, 2009; Tardito et al. 2011). These compounds typically act as lipid-soluble mobile carriers or hydrophobic channel formers (Ding and Lind 2009; Kevin Ii et al., 2009).

Several antimicrobial agents, including 8-hydroxyquinoline, thiosemicarbazones, and pyrithione, exhibit synergistic antimicrobial activity in the presence of copper (Navarro et al., 2019; Spoladori et al., 2023; Tardito et al. 2011). These compounds function as hydrophobic ionophores that compromise membrane integrity while simultaneously delivering cytotoxic copper ions into cells, resulting in potent antimicrobial effects (Ding and Lind 2009; Kevin Ii et al., 2009; Navarro et al., 2019; Spoladori et al., 2023; Tardito et al. 2011). Copper ionophores such as dithiocarbamates are lipophilic ligands that binds to Cu(I) or Cu(II) to form charge neutral hydrophobic complexes, allowing diffusion of copper ions across membrane (Ding and Lind 2009; Oliveri 2022; Tardito et al. 2011). These copper complexed lipophilic ligands disrupt membranes indirectly by promoting intracellular copper accumulation, which drives redox cycling and ROS formation, leading to lipid peroxidation. Consequently, membrane integrity, permeability barrier function, and cellular energy conservation are compromised. In addition, copper ionophores have been proposed to enhance the efficacy of copper-dependent cytotoxic mechanisms, including cuproptosis, in cancer therapy. Cuproptosis is a recently identified form of regulated cell death triggered by intracellular copper accumulation. In this process, copper binds to lipoylated mitochondrial proteins—primarily components of the tricarboxylic acid (TCA) cycle—inducing protein aggregation and destabilization of iron–sulfur cluster-containing proteins. These events lead to proteotoxic stress, mitochondrial dysfunction, and ultimately cell death (Oliveri 2022; Springer et al. 2024).

Although the precise mechanism of fluopsin C–mediated toxicity remains to be fully elucidated, three key observations support an ionophore-based model: (i) fluopsin C is a hydrophobic organocopper complex (Afonso et al., 2024; Del Rio et al. 1972; Egawa et al. 1970; Patteson et al. 2021), (ii) it exhibits broad, relatively nonselective toxicity against both prokaryotic and eukaryotic cells (Del Rio et al. 1972; Otsuka et al., 1972), and (iii) and affected cells exhibit damages in plasma membranes (Afonso et al., 2024; Alves de Lima et al., 2022; Ma et al. 2013; Navarro et al., 2019; Spoladori et al., 2023). Collectively, these properties suggest that fluopsin C may act as a copper-shuttling ionophore that disrupts membrane integrity, collapses membrane potential, and perturbs intracellular ionic balance through the translocation of toxic copper ions across cellular membranes.

## Fluopsin C production

Limited attempts have been made to optimize the production and purification of fluopsin C. In one study (Bedoya et al. 2019), a low-virulence (LV) strain of *Pseudomonas aeruginosa* isolated from citrus canker lesions of orange was evaluated using response surface methodology. Optimal fluopsin C production was achieved at a total copper concentration of 175 µM CuCl₂·2 H₂O (30 mg/L), comprising 5 mg/L present in the basic culture medium (BCM) and an additional 25 mg/L supplied during optimization. The BCM consisted of nutrient broth supplemented with 2 g/L peptone, 1.2 g/L meat extract, and 5 mg/L CuCl₂·2 H₂O. Maximum fluopsin C productivity was obtained using a 1% inoculum prepared from logarithmic-phase seed cultures (OD₅₉₀ = 0.09, approximately 1 × 10⁸ CFU/mL), followed by incubation at 28 °C under constant aeration for 10 days. Cultures were harvested when cell density reached an OD₅₉₀ of 0.12, yielding 11.11 ± 1.05 mg/L fluopsin C. Further increases in copper, meat extract, or peptone concentrations resulted in a substantial reduction in fluopsin C production. Notably, scale-up experiments revealed approximately a 20% decrease in productivity, with higher yields observed in 100 mL shake-flask cultures compared to 5 L fermenter systems.

In a separate study focused on characterizing fluopsin C biosynthesis (Patteson et al. 2021), a defined cultivation strategy was employed. *P. aeruginosa* PAO strains were grown in M9 minimal medium supplemented with trace elements at the following final concentrations: 50 µM (NH₄)₂Fe(SO₄)₂, 20 µM CaCl₂, 10 µM MnSO₄, 10 µM ZnSO₄, 2 µM CuSO₄, 2 µM NiCl₂, 2 µM (NH₄)₆Mo₇O₂₄, and 2 µM H₃BO₃. Seed cultures were prepared by overnight growth in Luria–Bertani broth, followed by centrifugation and washing twice with 200 mM MgCl₂. Washed cells were resuspended and used to inoculate 250 mL of M9 medium supplemented with trace elements. Fluopsin C production was induced by the addition of 125 µM CuSO₄ once cultures reached an OD₆₀₀ of 0.5–1.0. Induced cultures were incubated at 37 °C with agitation at 225 rpm. Although fluopsin C yield was not quantified in this study, the production strategy appears inherently more efficient.

Complex media such as nutrient broth contain chemically undefined organic components that can chelate free copper ions, thereby reducing copper bioavailability to *P. aeruginosa*. These copper-binding impurities may also complicate downstream recovery and purification of fluopsin C. In contrast, defined media such as M9 provide controlled chemical composition and predictable copper speciation, making them a superior choice for large-scale fluopsin C production.

## Conclusion

Copper lies at the core of a fundamental biological tradeoff: it is essential for redox biochemistry yet becomes rapidly cytotoxic when it escapes tight intracellular homeostasis control. The work summarized here emphasizes that copper toxicity is not simply a matter of metal overload, but a systems-level outcome driven by metalation, compartment-specific redox chemistry, and copper-enabled ROS formation through Fenton-like cycles. *Pseudomonas aeruginosa* exemplifies an organism that has evolved unusually robust, multi-layered defenses—deploying periplasmic surveillance and export (CopRS–PcoBA), cytoplasmic sensing and efflux (CueR-regulated CopA/CzcR regulated CzcCBA system), and an additional small-molecule strategy in which copper is neutralized by sequestration into the organometallic complex fluopsin C. The fluopsin C may be transported from the cell via a putative multidrug efflux pump MexPQ–OpmE.

In addition to its native role in maintaining copper homeostasis, fluopsin C represents a potential alternative antimicrobial agent because it exploits metal intoxication and membrane disruption rather than highly specific molecular targets, thereby circumventing many conventional antibiotic resistance mechanisms. As a copper-containing organometallic compound, fluopsin C exhibits broad-spectrum activity against Gram-positive and Gram-negative bacteria, fungi, and even cancer cells, consistent with a mechanism centered on membrane damage and ionophore-like copper shuttling. This mode of action is inherently difficult for microbes to evade through single-gene mutations and appears particularly effective against multidrug-resistant and biofilm-associated pathogens, especially when used in combination with membrane-active antibiotics such as polymyxins. However, a major limitation of fluopsin C has historically been its nonspecific cytotoxicity toward eukaryotic cells, which precludes routine systemic use. Consequently, the most realistic therapeutic applications of fluopsin C lie in localized or formulation-controlled contexts, including topical therapies, anti-biofilm adjunctive treatments, and encapsulated delivery systems designed to expand its therapeutic window. Although fluopsin C has been recently revisited because of its strong translational potential, efforts to optimize its production remain limited; therefore, future studies aimed at defining optimal cultivation conditions and improving productivity will be essential to advance its practical application.

## Data Availability

No datasets were generated or analysed during the current study.
